# JPEG and raw image files compared to direct measurement of the breast region ^1^

**DOI:** 10.1590/s0102-865020200100000008

**Published:** 2020-11-30

**Authors:** Paulo Rogerio Quieregatto, Miguel Sabino, Fabianne Furtado, Andrea do Amaral Quieregatto, Thales Waltenior Trigo, Lydia Masako Ferreira

**Affiliations:** I PhD, Postgraduate Program in Translational Surgery and Plastic Surgery Division, Universidade Federal de São Paulo (UNIFESP), Brazil. Analysis of data, manuscript preparation and writing, final approval.; II MS, PhD, Postgraduate Program in Translational Surgery and Plastic Surgery Division, UNIFESP, Sao Paulo-SP, Brazil. Analysis of data, manuscript preparation, final approval.

**Keywords:** Photogrammetry, Anthropometry, Software, Breast

## Abstract

**Purpose:**

To compare JPEG and RAW image file extensions to direct measurement of the breast region.

**Methods:**

Points were marked on the breasts and arms of 40 female volunteers. The joining of these points in each hemibody formed seven linear segments, one angular segment and one median segment common to both hemibodies. Volunteers were photographed in a standardized fashion and evaluated by three raters using the software Adobe Photoshop CS6^®^ and three image file extensions (RAW, high resolution JPEG and low resolution JPEG); values were compared to direct anthropometry.

**Results:**

All variables had interclass correlation coefficient higher than 0.8 (ICC>0.8). On average, all variables in all methods showed differences (p<0.05) when compared to direct measurement. A formula was created for each segment and each image file extension in comparison with the direct measurement.

**Conclusion:**

Measurements were similar among the correlated JPEG and RAW image file extensions but differed from the actual breast measurement obtained with a caliper.

## Introduction

Learning about all details and peculiarities of breasts is important for surgical programming and for determining possible limitations on the available operatory techniques that are presented to patients ^1^ . Knowing such limitations may prevent patients from creating unreal expectations about the proposed procedures ^2^ .

Breast evaluation by direct anthropometry requires considering chest mobility during respiration, besides protuberances and curves in the cutaneous tissue. Thus, in theory, indirect anthropometry of the breast region can become technically superior to direct anthropometry since measurements are obtained from a motionless image ^3^ ^,^ ^4^ .

Quieregatto *et al* . ^5^ evaluated female breast measurements using direct anthropometry and the comparison of three software types. Equal segments had discrepant measurements when analyzed with different tools, i.e., different complexity software types. The authors questioned whether the used file type, JPEG, could have influenced such discrepancy.

JPEG (Joint Photographics Experts Group) is the name of the group responsible for creating this method, which is used to compress photographic images, as a file format, obtaining a small image of moderate quality as the final result. The quality of a JPEG image is related to its size; thus, this format differs from the others for facilitating file storage and distribution. The compression level can be adjusted: the more the file is compressed, the smaller its size, but this implies image quality loss. One same JPEG image loses quality every time it is saved, since the file saving process of this method implies compression and consequently quality loss.

The RAW format is a generic denomination for digital image file formats containing the total data of the image as it was captured by the photographic camera sensor. Such formats cannot be compressed with information loss, as is the case for the popular JPEG. The raw format contains all data of the image captured by the camera and a greater color depth, in general 30 or 36 bits/pixel; thus, its files are very large, except when they are compressed (without losses). This format is accepted by the Brazilian Court of Justice as a proof. Each company names the RAW file differently. In the case of Nikon^®^, the RAW file is named NEF.

There are no studies in the literature comparing measurements obtained by different image file types (JPEG and RAW) or the quality of high and low resolution JPEG images.

Thus, the aim of the present study is to compare JPEG and RAW image file extensions with direct measurement of the breast region.

## Methods

The present study is primary, analytical, clinical, observational and transversal and was approved by the Research Ethics Committee, Universidade Federal de São Paulo (UNIFESP), under the number 430.239.

**Figure 1:**
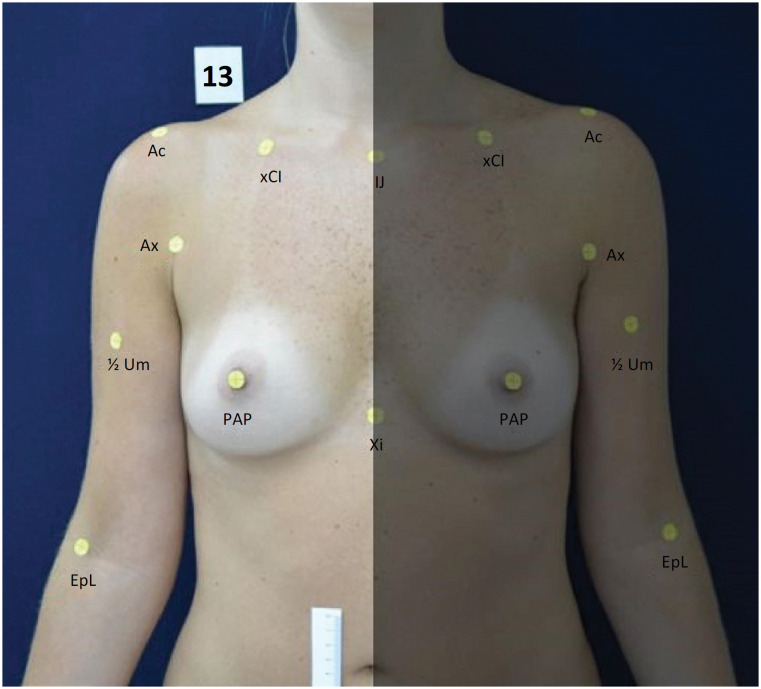
Anthropometric and anatomic points marked with tags.

Participants were 40 female volunteers aged between 18 and 60 years old (mean of 29 years and 10 months; standard deviation of 10 years and 3 months). Uni- or bilaterally mastectomized volunteers were excluded; and those showing *pectus carinatum* or *escavatum* -type thoracic deformities or ptosis surpassing the inferior transversal limit of the belly scar line, measured at pre-defined points ( Fig. 1 ), were also excluded.

Counterclockwise: IJ = center of jugular notch; xCl = midpoint between IJ and acromion; Ac = lateral prominence of the acromion; Ax = proximal point to the anterior axillary line; 1/2Um = midpoint between Ac and EpL; EpL = anterior projection of the lateral epicondyle; PAP = center of the mammary papilla; Xi =base of the xiphoid process.

**Figure 2:**
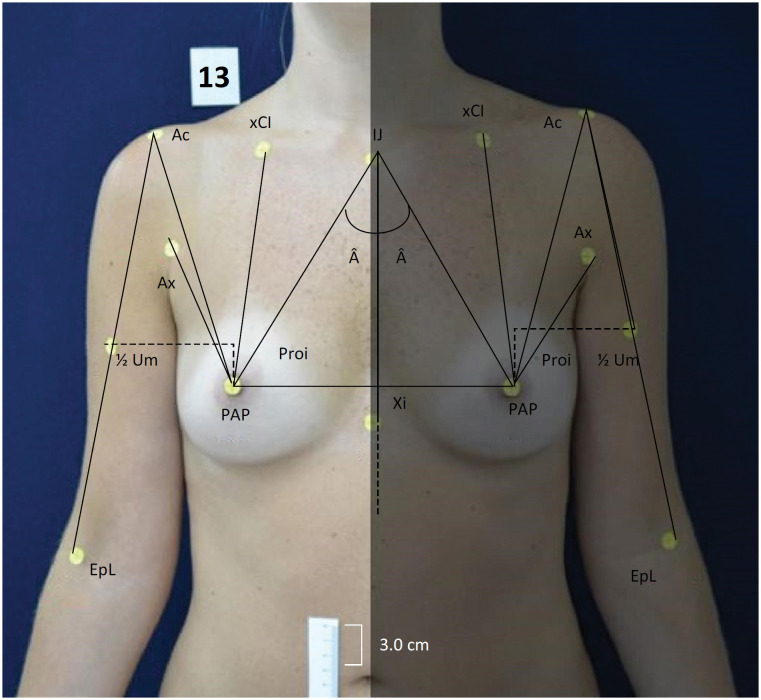
Schematic representation of the 17 segments formed by the joining of the adopted points, 9 linear segments and 1 angular measurement for each hemibody.

These points allowed the formation of 15 segments distributed on each hemibody, consisting of seven linear measurements and one angular measurement for each side ( Fig. 2 ).

Description of segments: IJ-Xi = center of the jugular notch to the base of the xiphoid process; IJ – PAP = center of the jugular notch to the center of the mammary papilla; xCl – PAP = Midpoint between the center of the jugular notch and acromion to the center of the mammary papilla; Ac –PAP = Lateral prominence of the acromion to the center of the mammary papilla; Ax- PAP = Proximal point to the anterior axillary line to the center of the mammary papilla; LM – PAP = Median anterior line to the center of the mammary papilla; Ac – EpL = Lateral prominence of the acromion to the anterior projection of the lateral epicondyle; Ac – ½ Um = Midpoint between the lateral prominence of the acromion to the anterior projection of the lateral epicondyle; Angle formed by the confluence of segments IJ-Xi and IJ-PAP; Proj = Projection of ½ Um on the ipsilateral mammary papilla line.

### Photographic standardization

A digital camera Nikon^®^, D3200^®^ model, 24.2 megapixels, 18–55-mm lenses and JPEG + RAW formatting, was used. All photographs were standardized to 16 megapixels, without optical zoom, and 50-mm lenses.

The photograph was taken in JPEG + NEF (RAW) mode, and the image was separated in these two file formats by the camera itself.

Photogrammetry was done by three independent researchers and the major researcher performed a second evaluation at 90 days after the first one to allow an intra-rater analysis. All three raters used the same computer to make measurements and received specific training for using the adopted software Adobe Photoshop CS6^®^.

The obtained digital photographs were inserted in the software, divided into three distinct file extensions: NEF, high resolution JPEG (on-board) and low resolution JPEG (off-board), and analyzed separately. The software tools were calibrated to the 3-cm measurement obtained from the numeral scale of the ruler attached to the right mesogastric region of the volunteer so that actual measurements could be obtained.

Liner measurements were collected once by Rater 1 using a caliper and transferred to a ruler. For angular measurements, a protractor was used.

### Linear regression

The obtained data underwent linear regression, which is related to the capability of predicting a value based on another known value (predicting Y as long as X is known, where X is the independent variable and Y is the dependent variable). In the studied case, Y corresponds to the variables in the methods NEF Photogrammetry, JPEG Photogrammetry on-board and JPEG Photogrammetry off-board, while X corresponds to the standard data of the direct measurement obtained with a caliper. Regression line equations were obtained for each variable as a function of the standard caliper. Thus, the value of the variable can be mathematically obtained for each value of the standard.

The concordance/reproducibility of variables was conducted by applying the intra-rater Intraclass Correlation Coefficient (ICC) for each method and inter-rater ICC for each method, in groups of 2 raters and among the 3 raters.

## Results

The obtained results were divided into the following subtypes:

*Intra-rater intraclass correlation coefficient for each method (NEF photogrammetry, JPEG photogrammetry on-board, low JPEG photogrammetry off-board). Concordance/reproducibility for Rater 1's intra-measurements*

All variables have Intraclass Correlation Coefficient higher than 0.8 (ICC > 0.8), which indicates a high correlation among the performed measurements. JPEG photogrammetry off-board is the most reproducible method since it has greater quantity of variables showing the highest ICC values, followed by NEF photogrammetry and, lastly, JPEG photogrammetry on-board ( Table 1 ).

**Table 1:** Evaluation of Rater 1's intra-measurements.

	Variable	ICC	CI (95%)		Absolute variation	
			Inferior	Superior	Mean	SD
NEF Photogrammetry	IJ_PAP	0.992	0.988	0.995	37.47	4.63
	xCI_PAP	0.995	0.992	0.997	34.05	4.85
	Ac_PAP	0.992	0.987	0.995	36.38	4.67
	Ax_PAP	0.997	0.995	0.998	21.60	4.07
	LM_PAP	0.991	0.987	0.995	20.66	2.71
	Ac_Epl	0.963	0.942	0.976	56.27	2.89
	Ac_1/2Um	0.97	0.953	0.980	28.45	1.69
	Projection	0.999	0.999	0.999	8.09	5.34
	Angle	0.933	0.895	0.957	67.18	7.30
JPEG Photogrammetry On-board	IJ_Xi	0.994	0.991	0.996	32.00	2.64
	IJ_PAP	0.997	0.995	0.998	37.49	4.60
	xCI_PAP	0.994	0.990	0.996	34.00	4.88
	Ac_PAP	0.945	0.914	0.964	36.46	5.04
	Ax_PAP	0.989	0.982	0.993	21.60	4.14
	LM_PAP	0.996	0.994	0.998	20.68	2.68
	Ac_Epl	0.976	0.962	0.984	56.24	3.05
	Ac_1/2Um	0.974	0.959	0.983	28.43	1.71
	Projection	0.997	0.996	0.998	8.05	5.34
	Angle	0.845	0.758	0.901	67.18	7.27
JPEG Photogrammetry Off-board	IJ_Xi	0.997	0.996	0.998	32.04	2.60
	IJ_PAP	0.996	0.994	0.998	37.48	4.63
	xCI_PAP	0.987	0.979	0.991	34.04	4.88
	Ac_PAP	0.999	0.998	0.999	36.43	4.68
	Ax_PAP	0.992	0.987	0.995	21.66	4.13
	LM_PAP	0.998	0.997	0.999	20.70	2.68
	Ac_Epl	0.990	0.984	0.993	56.31	3.09
	Ac_1/2Um	0.994	0.991	0.996	28.48	1.70
	Projection	0.956	0.932	0.972	8.23	5.36
	Angle	0.876	0.807	0.921	67.26	7.38

IJ = center of jugular notch; xCl = midpoint between IJ and acromion; Ac = lateral prominence of the acromion; Ax = proximal point to the anterior axillary line; 1/2Um = midpoint between Ac and EpL; EpL = anterior projection of the lateral epicondyle; PAP = center of the mammary papilla; Xi = base of the xiphoid process; Fotog = photogrammetry; NEF photogrammetry = RAW file from Nikon^®^ camera; JPEG photogrammetry on-board = Joint Point Expert Groups file converted inside the camera; JPEG photogrammetry off-board = Joint Point Expert Groups file converted by the software. 5% significance level (p<0.05). Intraclass correlation coefficient (ICC) was used.

*Inter-rater intraclass correlation coefficient for each method (NEF photogrammetry, JPEG photogrammetry on-board, Low JPEG photogrammetry off-board). Concordance/reproducibility among Raters 1, 2 and 3*

All variables have intraclass correlation coefficient higher than 0.8 (ICC > 0.8), which means a high correlation among the performed measurements. JPEG photogrammetry on-board is the most reproducible method since it has greater quantity of variables showing the highest ICC values, followed by JPEG photogrammetry on-board and, lastly, NEF photogrammetry ( Table 2 ).

**Table 2:** Inter-rater evaluation for Raters 1, 2 and 3.

	Variable	ICC	CI (95%)		Absolute variation	
			Inferior	Superior	Mean	SD
NEF Photogrammetry	IJ_Xi	0.992	0.988	0.994	48.22	3.98
	IJ_PAP	0.909	0.867	0.938	56.25	7.23
	xCI_PAP	0.997	0.995	0.998	51.33	7.29
	Ac_PAP	0.996	0.994	0.997	54.93	7.00
	Ax_PAP	0.996	0.994	0.997	32.56	6.05
	LM_PAP	0.990	0.985	0.993	31.09	4.06
	Ac_Epl	0.976	0.965	0.984	84.86	4.32
	Ac_1/2Um	0.981	0.972	0.987	42.95	2.49
	Projection	0.993	0.990	0.995	12.25	8.02
	Angle	0.935	0.905	0.956	101.18	10.93
JPEG Photogrammetry On-board	IJ_Xi	0.992	0.988	0.994	48.13	3.91
	IJ_PAP	0.993	0.989	0.995	56.31	6.81
	xCI_PAP	0.998	0.996	0.998	51.18	7.25
	Ac_PAP	0.997	0.996	0.998	54.75	7.00
	Ax_PAP	0.998	0.998	0.999	32.45	6.07
	LM_PAP	0.993	0.990	0.995	31.10	4.05
	Ac_Epl	0.985	0.978	0.990	84.61	4.59
	Ac_1/2Um	0.981	0.972	0.987	42.85	2.55
	Projection	0.990	0.985	0.993	12.28	7.98
	Angle	0.927	0.894	0.951	101.10	11.15
JPEG Photogrammetry Off-board	IJ_Xi	0.984	0.976	0.989	48.39	4.03
	IJ_PAP	0.989	0.984	0.993	56.54	6.76
	xCI_PAP	0.994	0.991	0.996	51.34	7.20
	Ac_PAP	0.996	0.994	0.997	55.01	6.91
	Ax_PAP	0.999	0.998	0.999	32.60	6.07
	LM_PAP	0.994	0.991	0.996	31.21	4.06
	Ac_Epl	0.985	0.978	0.990	84.91	4.66
	Ac_1/2Um	0.877	0.821	0.917	43.13	2.41
	Projection	0.996	0.995	0.998	12.24	8.03
	Angle	0.894	0.846	0.929	100.48	11.35

IJ = center of jugular notch; xCl = midpoint between IJ and acromion; Ac = lateral prominence of the acromion; Ax = proximal point to the anterior axillary line; 1/2Um = midpoint between Ac and EpL; EpL = anterior projection of the lateral epicondyle; PAP = center of the mammary papilla; Xi = base of the xiphoid process; Fotog = photogrammetry; NEF photogrammetry = RAW file from the Nikon^®^ camera; JPEG photogrammetry on-board = Joint Point Expert Groups file converted inside the camera; JPEG photogrammetry off-board = Joint Point Expert Groups file converted by the software. 5% significance level (p<0.05). Intraclass correlation coefficient (ICC) was used.

*Intraclass correlation coefficient for each method (NEF photogrammetry, JPEG photogrammetry on-board, low JPEG photogrammetry off-board) compared to direct measurements (caliper)*

Except for the variables “Ac_1/2Um” and “Angle” in the method NEF photogrammetry and the variable “Angle” in the method JPEG photogrammetry off-board, the remaining variables have intraclass correlation coefficient higher than 0.8 (ICC > 0.8), which represents a high correlation among the performed measurements. Lower ICC values indicate a lower correlation, but still showing good index. JPEG photogrammetry off-board is the most reproducible method since it shows greater quantity of variables with the highest ICC values, followed by JPEG photogrammetry on-board and, lastly, NEF photogrammetry ( Table 3 ).

**Table 3:** Evaluation of each method compared to direct measurement.

	Variable	ICC	CI (95%)		Absolute variation	
			Inferior	Superior	Mean	SD
NEF Photogrammetry	IJ_Xi	0.954	0.928	0.970	33.42	2.67
	IJ_PAP	0.975	0.961	0.984	39.45	4.72
	xCI_PAP	0.969	0.952	0.980	37.35	4.98
	Ac_PAP	0.934	0.897	0.958	41.41	4.66
	Ax_PAP	0.916	0.869	0.946	25.32	4.50
	LM_PAP	0.956	0.931	0.972	20.95	2.62
	Ac_Epl	0.908	0.857	0.941	58.54	2.63
	Ac_1/2Um	0.799	0.687	0.871	29.44	1.37
	Projection	0.968	0.950	0.980	9.04	5.60
	Angle	0.769	0.640	0.852	66.23	6.75
JPEG Photogrammetry On-board	IJ_Xi	0.948	0.918	0.966	33.42	2.67
	IJ_PAP	0.974	0.959	0.983	39.45	4.70
	xCI_PAP	0.967	0.948	0.979	37.35	4.99
	Ac_PAP	0.931	0.892	0.956	41.40	4.69
	Ax_PAP	0.907	0.854	0.940	25.32	4.49
	LM_PAP	0.967	0.948	0.979	20.96	2.61
	Ac_Epl	0.939	0.905	0.961	58.53	2.81
	Ac_1/2Um	0.860	0.781	0.910	29.42	1.44
	Projection	0.966	0.948	0.978	9.05	5.60
	Angle	0.787	0.668	0.863	65.97	6.83
JPEG Photogrammetry Off-board	IJ_Xi	0.957	0.933	0.973	33.48	2.69
	IJ_PAP	0.977	0.964	0.985	39.51	4.70
	xCI_PAP	0.969	0.952	0.980	37.38	4.98
	Ac_PAP	0.936	0.900	0.959	41.45	4.67
	Ax_PAP	0.911	0.861	0.943	25.33	4.49
	LM_PAP	0.966	0.948	0.978	20.99	2.62
	Ac_Epl	0.930	0.891	0.955	58.58	2.83
	Ac_1/2Um	0.849	0.765	0.903	29.47	1.44
	Projection	0.968	0.950	0.980	9.06	5.59
	Angle	0.786	0.667	0.863	66.01	6.90

IJ = center of jugular notch; xCl = midpoint between IJ and acromion; Ac = lateral prominence of the acromion; Ax = proximal point to the anterior axillary line; 1/2Um = midpoint between Ac and EpL; EpL = anterior projection of the lateral epicondyle; PAP = center of the mammary papilla; Xi = base of the xiphoid process; Fotog = photogrammetry; NEF photogrammetry = RAW file from Nikon^®^ camera; JPEG photogrammetry on-board = Joint Point Expert Groups file converted inside the camera; JPEG photogrammetry off-board = Joint Point Expert Groups file converted by the software. 5% significance level (p<0.05). Intraclass correlation coefficient (ICC) and linear regression equations through Pearson's correlation index were used.

*Description of absolute differences between each method and direct measurement for each evaluated segment, and results of comparisons of such differences between methods*

On average, all variables in all methods had differences (p < 0.05) when compared with those obtained by direct measurement with a caliper ( Table 4 ).

**Table 4:** Evaluation of each method compared to direct measurement for each evaluated segment.

Variable	Method	Mean	SD	Minimum	Maximum	*p*
IJ_Xi	NEF Photogrammetry	1.44	0.57	1.313	1.569	0.000
	JPEG Photogrammetry On-board	1.44	0.61	1.308	1.580	0.000
	JPEG Photogrammetry Off-board	1.38	0.56	1.255	1.503	0.000
IJ_PAP	NEF Photogrammetry	1.97	0.75	1.806	2.139	0.000
	JPEG Photogrammetry On-board	1.97	0.76	1.802	2.141	0.000
	JPEG Photogrammetry Off-board	1.91	0.72	1.750	2.069	0.000
xCI_PAP	NEF Photogrammetry	3.28	0.88	3.084	3.475	0.000
	JPEG Photogrammetry On-board	3.28	0.91	3.079	3.483	0.000
	JPEG Photogrammetry Off-board	3.25	0.87	3.052	3.441	0.000
Ac_PAP	NEF Photogrammetry	4.98	1.19	4.717	5.248	0.000
	JPEG Photogrammetry On-board	4.99	1.24	4.717	5.266	0.000
	JPEG Photogrammetry Off-board	4.94	1.18	4.680	5.207	0.000
Ax_PAP	NEF Photogrammetry	3.69	1.31	3.403	3.984	0.000
	JPEG Photogrammetry On-board	3.70	1.37	3.390	4.001	0.000
	JPEG Photogrammetry Off-board	3.68	1.34	3.382	3.978	0.000
LM_PAP	NEF Photogrammetry	0.29	0.55	0.164	0.410	0.000
	JPEG Photogrammetry On-board	0.28	0.47	0.176	0.388	0.000
	JPEG Photogrammetry Off-board	0.25	0.48	0.141	0.355	0.000
Ac_Epl	NEF Photogrammetry	2.27	0.80	2.092	2.446	0.000
	JPEG Photogrammetry On-board	2.27	0.70	2.119	2.428	0.000
	JPEG Photogrammetry Off-board	2.22	0.75	2.057	2.390	0.000
Ac_1/2Um	NEF Photogrammetry	0.96	0.61	0.825	1.097	0.000
	JPEG Photogrammetry On-board	0.98	0.54	0.857	1.096	0.000
	JPEG Photogrammetry Off-board	0.93	0.56	0.805	1.053	0.000
Projection	NEF Photogrammetry	1.00	1.00	0.773	1.218	0.000
	JPEG Photogrammetry On-board	0.98	1.03	0.751	1.208	0.000
	JPEG Photogrammetry Off-board	0.98	1.00	0.753	1.198	0.000
Angle	NEF Photogrammetry	-1.18	3.24	-1.896	-0.454	0.002
	JPEG Photogrammetry On-board	-0.92	3.15	-1.625	-0.223	0.010
	JPEG Photogrammetry Off-board	-0.96	3.19	-1.664	-0.246	0.009

IJ = center of jugular notch; xCl = midpoint between IJ and acromion; Ac = lateral prominence of the acromion; Ax = proximal point to the anterior axillary line; 1/2Um = midpoint between Ac and EpL; EpL = anterior projection of the lateral epicondyle; PAP = center of the mammary papilla; Xi = base of the xiphoid process; Fotog = photogrammetry; NEF photogrammetry = RAW file from Nikon^®^ camera; JPEG photogrammetry on-board = Joint Point Expert Groups file converted inside the camera; JPEG photogrammetry off-board = Joint Point Expert Groups file converted by the software; *p* = evaluated by Bonferroni multiple comparisons. 5% significance level (p<0.05). Repeated measures ANOVA was used.

### Linear regression

Results of the evaluated data related to linear regression of the obtained measurements.

As the obtained values were constant, a formula could be found for each segment and each image file extension, compared to the direct measurement ( Table 5 ).

**Table 5:** Linear regression equations for each method and direct measurement for each segment and different evaluated files.

SEGMENT	NEF	JPEG ON-BOARD	JPEG OFF-BOARD
IJ - Xi	Y = 0,43 + 0.57X or X = (Y – 0,43) / 0.57	Y = 0.71 + 0.92X or X = (Y – 0.71) / 0.92	Y = 0.57 + 0.93X or X = (Y – 0.57) / 0.93
IJ - PAP	Y = 0.01 + 0.95X or X = (Y – 0.01) / 0.95	Y = 0.11 + 0.95X or X = (Y – 0.11) / 0.95	Y = 0.09 + 0.95X or X = (Y – 0.09) / 0.95
xCl - PAP	Y = 0.33 + 0.94X or X = (Y – 0.33) / 0.94	Y = 0.33 + 0.94X or X = (Y – 0.33) / 0.94	Y = 0.3 + 0.93X or X = (Y – 0.3) / 0.93
Ac -PAP	Y = 0.45 + 0.91X or X = (Y – 0.45) / 0.91	Y = 0.57 + 0.92X or X = (Y – 0.57) / 0.92	Y = 0.49 + 0.91X or X = (Y – 0.49) / 0.91
Ax- PAP	Y = 0.49 + 0.84X or X = (Y – 0.49) / 0.84	Y = 0.56 + 0.83X or X = (Y – 0.56) / 0.83	Y = 0.55 + 0.84X or X = (Y – 0.55) / 0.84
LM - PAP	Y = 0.08 + 0.98X or X = (Y – 0.08) / 0.98	Y = 0.06 + 0.98X or X = (Y – 0.06) / 0.98	Y = 0.06 + 0.98X or X = (Y – 0.06) / 0.98
Ac - EpL	Y = 1.42 + 0.92X or X = (Y – 1.42) / 0.92	Y = 0.93 + 0.99X or X = (Y – 0.93) / 0.99	Y = 0.95 + 0.99X or X = (Y – 0.95) / 0.99
Ac – ½ Um	Y = 1.1 + 0.9X or X = (Y – 1.1) / 0.9	Y = 0.05 + 0.97X or X = (Y – 0.05) / 0.97	Y = 0.04 + 0.97X or X = (Y – 0.04) / 0.97
Projection	Y = 0.1 + 0.92X or X = (Y – 0.1) / 0.92	Y = 0.08 + 0.92X or X = (Y – 0.08) / 0.92	Y = 0.08 + 0.92X or X = (Y – 0.08) / 0.92
Â	Y = 8.2 + 0.83X or X = (Y – 8.2) / 0.83	Y = 5.57 + 0.84X or X = (Y – 5.57) / 0.84	Y = 5.37 + 0.85X or X = (Y – 5.37) / 0.85

IJ = center of jugular notch; xCl = midpoint between IJ and acromion; Ac = lateral prominence of the acromion; Ax = proximal point to the anterior axillary line; 1/2Um = midpoint between Ac and EpL; EpL = anterior projection of the lateral epicondyle; PAP = center of the mammary papilla; Xi = base of the xiphoid process; Fotog = photogrammetry; NEF photogrammetry = RAW file from Nikon^®^ camera; JPEG photogrammetry on-board = Joint Point Expert Groups file converted inside the camera; JPEG photogrammetry off-board = Joint Point Expert Groups file converted by the software; Y = Measurement obtained by the software; X = Direct measurement obtained with a caliper.

## Discussion

Learning more about female breasts can improve the understanding of women's physical and mental health ^6^ . The first to evaluate the breast by direct anthropometry was Penn in 1955 ^7^ . Since then, different authors have published studies aimed at developing protocols for breast measurement by direct anthropometry and at highlighting the limitations of such measurements ^4^ ^,^ ^8^ ^–^ ^14^ .

Odo *et al* . ^15^ analyzed pre- and post-operatory results of breast asymmetry by direct anthropometry. In comparative studies of breast asymmetry corrective surgeries, Pozzobon *et al* . ^6^ used nuclear magnetic resonance of breasts and linear measurements.

For the thoracic region, measurements can be less accurate due to the several curves, depressions and protuberances not only in the female breasts, but also in the thoracic region. Besides, the thoracic wall mobility during respiration can oscillate, not only among individuals but also in one same individual for measurements taken at different times ^4^ ^,^ ^1^ ^2^ . The need of developing a method that could minimize such changes caused by the thorax wall mobility has led Quieregatto *et al* . ^13^ to determine, based on the studies by Penn ^7^ , Smith *et al* . ^8^ and Weistrech ^4^ , points in the thorax, according to anthropometric and anatomic points, which could allow standardized breast measurements ^16^ . The best applicability of indirect anthropometry compared to direct anthropometry has been discussed by the authors in previous studies. In indirect anthropometry, the measurements should be the same since they are obtained from an image, which is unchangeable, differently from direct anthropometry, in which measurements are performed directly on the body and may result in discrepancies due to possible changes in the body contour over time.

With the evolution of Informatics and the development of several graphics software types to measure diverse body parts, Sivagnanavel *et al* . ^17^ and Assunção *et al* . ^18^ proposed software validation studies, comparing the software types, since they use the same digital tool and have the same theoretical foundation but can yield different results. Software types of different complexities showed different results for breast measurement ^5^ .

Quieregatto *et al* . ^5^ developed a photographic standardization protocol for the breast region and compared indirect anthropometry (computerized photogrammetry using three software types) to direct anthropometry. Three different types of software were used: Image Tool^®^, Photoshop^®^ and Autocad^®^, which differed from each other in inter- and intra-rater measurements and when compared with the actual measurement obtained with a caliper. The present study adopted Adobe Photoshop CS6^®^ since it was the only software type that allowed studying RAW image files. The adopted methodology was based on these previous studies and the focal length was variable, depending only on the framing. New studies have already been conducted considering the difference between the lens and the object under analysis.

All measurements had ICC higher than 0.8, i.e., values were very close to 1.0, the ideal correlation, which indicates a high correlation among the measurements taken by the three raters and between the two measurements taken by one same rater at different times for RAW, JPEG on-board (high resolution) and JPEG off-board (low resolution). This indicates that all obtained measurements were similar. The only exception was “Ac_½Um” (distance between the acromion and the midpoint between the acromion and the anterior projection of the epicondyle); the value found by the first and second raters was different from that obtained by the third rater for low resolution JPEG file ( Table 3 ), which may evidence some limitation on the third rater's measurement of that segment, specifically for that specific image file. The absence of difference between measurements made with high and low resolution RAW and JPEG files of breast photographs leads to the conclusion that different files can be used to obtain this breast measurement, without loss in the image quality and consequent distortion of measurements.

Comparison of the values obtained by the software to the actual measurement for the different image files indicated a p < 0.05, which means that the indirect anthropometric measurements are different from the actual ones. This finding corroborates the results obtained by Quieregatto *et al* . ^5^ who reported differences between direct and indirect measurements of the breasts.

Although there is some correspondence between direct anthropometry and photogrammetry for some body parts (head, face, eyes, nose, mouth and ears) ^12^ , studies of the breast region conducted by Quieregatto *et al* . ^5^ did not demonstrate an effective formula to identify the actual measurement based on measurements obtained by indirect anthropometry. The present study was intended to understand the discrepancies found in previous studies and propose a safe manner to measure breasts by photogrammetry, assuring analysis and reproducibility.

In the evaluation of the different image files for breast measurements according to the parameters suggested by Quieregatto *et al* . ^5^ , Adobe Photoshop CS6^®^ showed that both inter- and intra-rater measurements were concordant. This finding evidences that the adopted software has high liability, reproducibility and consequently applicability in the clinical practice as long as software operators receive a short training about its handling ^19^ . Thus, linear regression equations could be elaborated to learn about both direct measurement from indirect measurement and indirect measurement from previously known direct measurement. 2D and 3D breast anthropometric evaluations have been performed in new studies.

The differences between image files are relevant in research with JPEG and RAW files, evidencing their importance for breast evaluation by indirect anthropometry ^20^ . The present study also demonstrates that the three different image file extensions that were evaluated were similar and that breast photogrammetry comparison cannot be used indiscriminately. The type of equipment that will be used to obtain the images for analysis must be defined, especially in studies involving two distinct breast evaluation times, such as pre- and post-operatory evaluations; in this case, the same piece of equipment should be employed in both study times.

## Conclusions

Measurements were similar among all three image file extensions as JPEG and RAW. The actual measurement of breasts, obtained with a caliper, was different from that obtained by the software.
